# Supplementary material for: Backtracking: Improved methods for identifying the source of a deliberate release of Bacillus anthracis from the temporal and spatial distribution of cases
Source: PLoS Comput Biol. 2024 Sep 6;20(9):e1010817. doi: 10.1371/journal.pcbi.1010817 (PMC11419379; doi:10.1371/journal.pcbi.1010817)
Supplement: S1 Supporting Information — Section A Grid Section parameter ranges, Section B Constructing RCNN inputs, Section C Predicted dose distribution. (PDF) [file pcbi.1010817.s001.pdf]

Supplementary Material

A Grid Search parameter ranges

Table A shows the parameter ranges used in the Grid Search method. For the time parameter, t_0 refers to the earliest symptom onset time in the observed data. The parameter range is separated into a grid of twenty equal steps, except for strength (S) which uses 21 steps.

Table A. Grid Search parameter ranges.

**Parameter**	**Minimum**	**Maximum**
(p_x, p_y)	$(62700, 8000)$	$(655400, 1216500)$
t	$t_0 - 13\,days$	t_0
$\log_{10}(S)$	10	20
U_s	0.1	10
W_d	0	360

B Constructing RCNN inputs

Input X_1 is constructed by splitting the spatial extent of the cases into an $N_x \times N_y$ grid, and the temporal extent into N_t time steps. Hence, the geographic extent covered by a single grid square (x_i, y_j) is given by

$$x_{min} + (i-1) \cdot \Delta_x < x_i \leq x_{min} + i \cdot \Delta_x \tag{1}$$

and

$$y_{min} + (j-1) \cdot \Delta_y < y_j \leq y_{min} + j \cdot \Delta_y, \tag{2}$$

for $i \in \{1, 2, ..., N_x\}$ and $j \in \{1, 2, ..., N_y\}$, where,

$$\Delta_x = \frac{x_{max} - x_{min}}{N_x} \tag{3}$$

and

$$\Delta_y = \frac{y_{max} - y_{min}}{N_y} \tag{4}$$

with $x_{min}, x_{max}, y_{min}$ and y_{max} representing the geographic bounds of cases in the outbreak in the original coordinate system used in the line list.

Similarly, the temporal discretisation can be represented as:

$$t_{min} + (k-1) \cdot \Delta_t < t_k < t_{min} + k \cdot \Delta_t \tag{5}$$

for $k \in \{1, 2, .., N_t\}$, and

$$\Delta_t = \frac{t_{max} - t_{min}}{N_t}. \tag{6}$$

At each time step t_k, we have three arrays of dimensions $N_x \times N_y$, representing the relative spatial distribution of new cases, hospitalisations and deaths during that time step. These values are normalised so that the each observation X_1^n has values between 0 and 1 through the application of a scaler ϕ_n.

The geographic and temporal context of X_1 is provided by the input X_2, which is constructed using the bounding data $x_{min}, x_{max}, y_{min}$, y_{max}, t_{mins} and t_{max}, as well as the scaler ϕ_n. In addition to this, X_2 also contains a first-guess of the angle of distribution of cases, θ, calculated as the arc-tangent of the coefficient of a linear model fitted through the case data. Finally, X_2 contains a further scaler, ψ, related to the population input, X_3, discussed below. All inputs in X_2 are standardized so that each feature has a mean of zero and standard deviation of one.

The final input into the model, X_3, describes the population distribution within the geographic bounds of X_1, using data provided by ONS. This is again normalised using a scaling factor ψ so that the values in each array are bounded between 0 and 1.

C Predicted dose distribution

Figures A-C shows the predicted plumes across the ten test outbreaks for the Grid-Search, MCMC and RCNN methods respectively. To produce these we use the Grid-Search, MCMC and RCNN methods to provide a predicted parameterisation for a given outbreak, then use the forward model to predict the atmospheric dispersion of anthrax for that parametirsation. In each case, we have plotted the contour encircling the 75th (dashed line) and 95th percentile (dashed line) of total dose. Both the true (green) and predicted (red) plumes are shown on each plot.

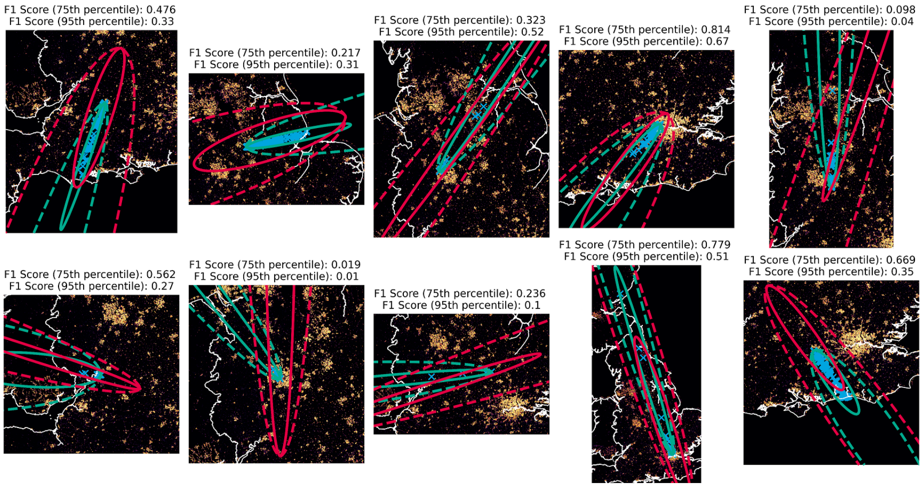

Fig A. Plumes predicted by the grid search method on the ten test datasets. The Green lines show the contours encircling the 75th (dashed line) and 95th percentile of total dose, the true values are illustrated by the red lines. Blue dots show the geographic location of cases. Relative population density is illustrated in orange. We have included the F1 scores, indicating the degree of overlap between areas covered by the true and predicted 75th percentile and 95th percentile contours.

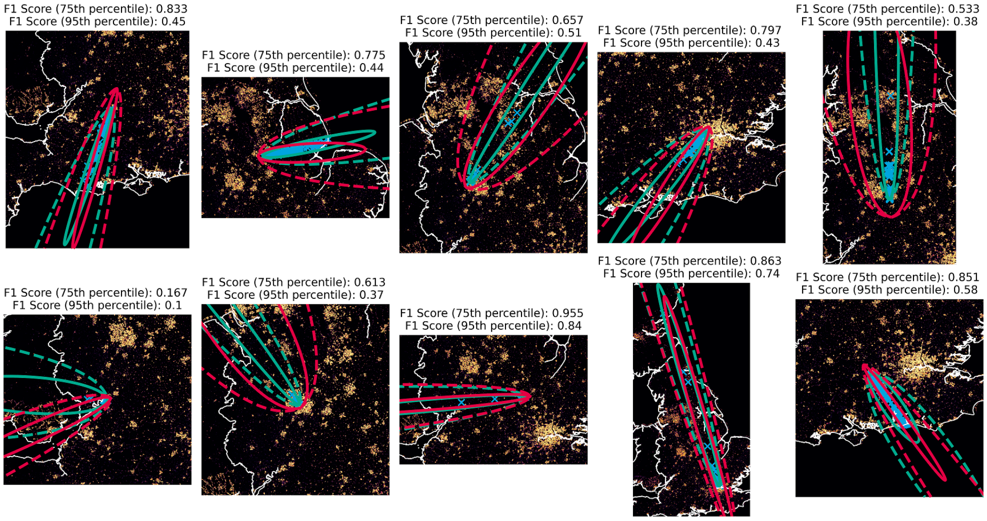

Fig B. Plumes predicted by the MCMC method on the ten test datasets. The Green lines show the contours encircling the 75th (dashed line) and 95th percentile of total dose, the true values are illustrated by the red lines. Blue dots show the geographic location of cases. Relative population density is illustrated in orange. We have included the F1 scores, indicating the degree of overlap between areas covered by the true and predicted 75th percentile and 95th percentile contours.

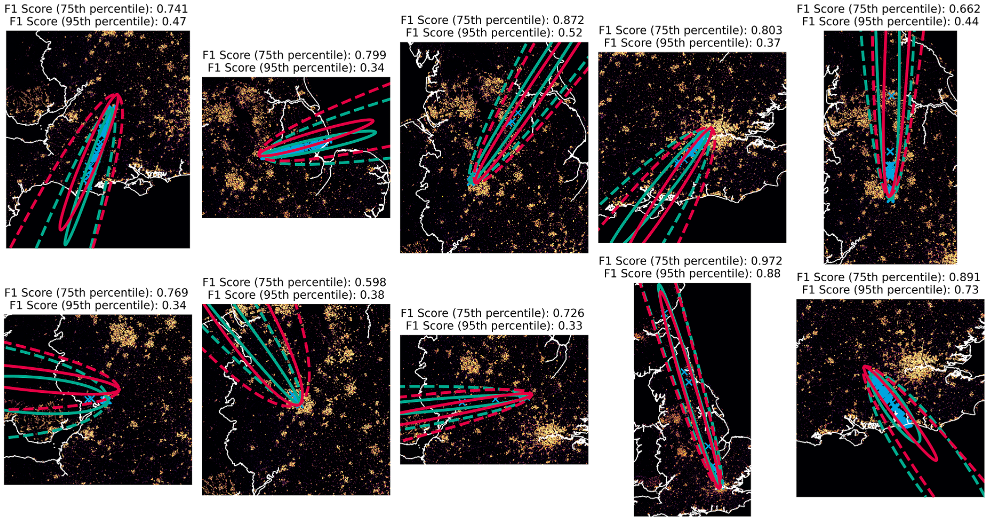

Fig C. Plumes predicted by the RCNN method on the ten test datasets. The Green lines show the contours encircling the 75th (dashed line) and 95th percentile of total dose, the true values are illustrated by the red lines. Blue dots show the geographic location of cases. Relative population density is illustrated in orange. We have included the F1 scores, indicating the degree of overlap between areas covered by the true and predicted 75th percentile and 95th percentile contours.
