# Supplementary material for: Backtracking: Improved methods for identifying the source of a deliberate release of Bacillus anthracis from the temporal and spatial distribution of cases
Source: PLoS Comput Biol. 2024 Sep 6;20(9):e1010817. doi: 10.1371/journal.pcbi.1010817 (PMC11419379; doi:10.1371/journal.pcbi.1010817)
Supplement: Attachment — Submitted filename: 2024-03-12 Coving letter post review.docx [file pcbi.1010817.s002.docx]

UK Health Security Agency

Manor Farm Road

Porton Down

SP4 0JG

Dear Dr Pitzer,

Thank you very much to you and your reviewers for your time and consideration of our manuscript. We have revised the manuscript in light of these comments and believe that this substantially improves the paper.

We include a point-by-point address to the reviewers’ comments below and reaffirm that this work is original and has not been published elsewhere, nor is it currently under consideration for publication elsewhere.

The authors continue to have no conflicts of interest to disclose.

Yours sincerely

Thomas Finnie

**Reviewers' comments:**

**Reviewer #1:**

1) *Line 22 - 24, 'two planar directions' reads oddly and planar later, perhaps 'across 2 dimensional plan is better'. Though the briggs model I think allows height as it is a Gaussian puff model with empirical diffusion terms.*

We agree and have replaced with the text "a two-dimensional plane representing ground level".

2) *Line 30 'realistic' may be too strong, it is certainly credible or plausible but the simplicity of Briggs given turbulent flow and population variation, with a lack of observational data of such an event means the simulation proposed is unvalidated. This does not undermine approach or results but may inflate readers expectations.*

Agree, have replaced with "reasonable".

3) *In intro the citation for within host model is [7] but in methods it becomes [8] can authors clarify.*

We believe [7] contains the incubation period model and [8] the symptom to hospitalisation and death models. This is clearer in the Supplement material. We have changed text to take this clearer and have also replaced [8] with Holty's 2006 paper which we believe is a better reference for the symptom to hospitalisation and death models. Have also expanded Supplement material to indicate which model in [7] is being used.

4) *Line 71 - reads oddly. "hospitalisations or deaths are recorded after the censor date, even if we know that" i think should be "hospitalisations or deaths are used after the censor date, even if we simulate that".*

We have replaced the text with "even if such events occur in the simulation"

5) *Line 75 - typo, no need for 'of'.*

We have deleted the word “of”.

6) *Line 135 - " guaranteed not" is too strong, I think should be "not guaranteed" was intended, unless the authors have a citation for a proof.*

Replaced with “but it will only find the global maxima in the highly unlikely situation that this falls at one of the grid points searched. Precise location of this maxima would generally require…”

7) *Figures 4, 5, 6 - in cation can the authors explain the symbols and lines.*

For Fig 4, 5 and 7 we have added “The different symbols represent the different test simulated outbreaks, the red dashed line is the predicted value equals true value line and in the last plot (wind direction), the black dashed line shows the line on which the predicted value differs from the true value by $\pm180^\circ$.” For Fig 6, added “Each circle represents a different test simulated outbreak, the red dashed line is the predicted value equals true value line and in the last plot (wind direction), the black dashed line shows the line on which the predicted value differs from the true value by $\pm180^\circ$.”

8) *Line 292 - "the preparation of synthetic training data," please add something like "and validity of simulation model" as method would be prone to structural misspecification.*

We have inserted “and validation” into “the preparation and validation of synthetic training data”.

9) *Line 311-12 - I agree but for grid search and MCMC the range of search/prior distribution definition may be considered given the structural assumptions in model to limit the range and chance of missing global maxima. This is going to potentially bias the results if the model is misspecified of course. Given the spatial pattern in Briggs is diffusion dominated after some distance most information is in the close cases - which if release was far from population centre may mean there is limited info in the data or likelihood may infer as may be equivalent to a far release. So work effort may be on the structure of the underlying model too.*

Lack of complete orthogonality between the model parameters and the varying information carried by each case is a reasonable point. We have expanded on this in the discussion a little.

**Reviewer #2**

1. *This paper has no literature review. As a result, it is completely impossible to assess whether or not what the authors are contributing is a valuable contribution. For example, I did a quick google scholar search with the following search terms: "neural network" and "outbreak source location", and saw a wide variety of papers. The geographic profiling literature has no doubt made contributions worth discussing as well. Additionally, the authors dedicate considerable time to discussing limitations to MCMC-based procedures, but don't review them meaningfully. There is an entire body of statistical literature that addresses the same limitations to metropolis-hastings style algorithms that the authors note, but seemingly fail to incorporate in their work. Basically, the authors are trying to argue that they've developed a novel tool that out-performs existing methods. However, by not providing a meaningful literature review, they cannot convince this reviewer that their contribution is novel.*

It is fair to say that we did not provide sufficient introduction to the subject in the original manuscript. We have provided a much more detailed introduction that aims to set this work in context for the reader both for the MCMC method and previous work on using neural networks for estimate parameters. We have not included work on geoprofiling as, while the underlying mathematics have been applied to epidemiological problems (including this problem, see Thompson J, *et al*) it does not cover either the computational or likelihood surface problems addressed here.

2. *In the section introducing forward models (and the Briggs model, specifically), the authors need to introduce this better in the main text instead of the appendix. I do not feel it is sufficient for future readers of this paper to simply say that they use the Briggs' model and then point to the appendix. I think the first page of the supplement would be better suited in the main text.*

The reviewer is correct. In our effort to not overburden the main text we removed too much technical detail. We have moved the first part of the supplementary material back to the main text and integrated it to aid the reader.

3. *The RCNN is poorly described. For example, what's an LSTM layer? It's never described.*

Agree. We have expanded the description of the RCNN and included references covering further details of CNN and LSTM in the relevant section.

4. *Results table 1. If these are R^2 values, why are some of them negative?*

We have expanded the description to “The coefficient of determination or $R^2$ score for each method across each parameter set is given in table1. When applied to non-linear regression, as here, the R^2 score can be negative. This occurs when the fit is in fact worse than the constant fit of the predicted value being taken as the mean of the training values. A positive value indicates that the fit is better than this constant fit with a maximum score value of 1 representing a perfect fit. For further details see for example Chicco et al.” We believe this covers the reviewer’s concerns.

5. *The entire results section is written as captions for figures and tables. Also, what are the criteria for a method being "good" or not having "a great deal of accuracy"?*

We have substantially reworded the results section and replaced the comparatives with R^2 values so that the reader may judge for themselves the accuracy and goodness of fit of each method. We believe that this significantly improves the section and thank the reviewer for a point well made.

6. *I simply don't understand the notion that releasing the code to this model is too much of security threat. In part this is because the authors have failed to convince me that they've made a novel contribution (see point 1 above). Do the authors think that if people who are prone to releasing anthrax in the UK had the code that detailed this modeling approach, they would alter their plans in any way? Not being an expert in this area, I assume their concerns are more focused on logistics than machine learning.*

We do understand the reviewer’s concern with regard to code release and, in general, we do strive to make public all the code used within our academic publications. We hope that with the revisions made to the manuscript that the problem we were hoping to address, and the solution proposed have shown to be more clearly novel and that by attempting to publish this work – even without code – we further human understanding of this and allied problems. We also understand that that while any individual contemplating direct participation in such an act is unlikely to be interested in the code, indeed as the reviewer notes having greater logistical concerns to surmount, such criminality is rarely perpetrated in isolation (see Takahashi H, et al. ) and the forward model code used here would likely to be of significant interest to individuals engaged in the wider support and planning of the act itself.

1.

**Thompson J, *et al.*** *Catching clouds: Simultaneous optimization of the parameters of biological agent plumes using Dirichlet processes to best estimate infection source location*. *2017 Federated Conference on Computer Science and Information Systems (FedCSIS)*. 2017, p. 481–484.

1.

**Takahashi H, *et al.*** Bacillus anthracis Bioterrorism Incident, Kameido, Tokyo, 1993. *Emerging Infectious Diseases* Centers for Disease Control and Prevention, 2004; **10**: 117.
